# Case of Restoration of the Perineum

**Published:** 1874-02-15

**Authors:** J. T. Everett

**Affiliations:** Sterling, Ill.


### CASE OF RESTORATION OF THE PERINEUM.

By J. T. Everett, M.D., Sterling, Ill.

WAS called, Sept. 20th, to see Mrs S., who had been confined, three days previous, under the care of another physician. The labor was said to have been severe and protracted.

The patient being a primipara, complete laceration of the perineum had resulted from the inelasticity of the soft parts. Found patient suffering intense agony; pulse 150 per minute; tongue furred and dry; temperature 103°; bowels tympanitic; and complete suppression of the urine, with all the symptoms of an advanced stage of peritonitis.

I immediately put the patient on half grain doses of morphia sulph., alternating with the following:

℞.—Quinia sulph., ʒ ss.

Tinct. verat viride, ʒ j.

Carbol. acid (95 per cent.), ʒ ss.

Aqua camph., ℥ ij.

M. S.—Teaspoonful every half hour.

Upon examination, found the labia of a dark congested hue, and emitting a foul odor, as though putrefactive changes had already commenced taking place; and the abdomen of a deep red hue, showing the extent of the peritonitis.

Thinking the case a desperate one, I held out but little hopes of recovery for the patient, but told the friends I would do all I could for her. I caused cloths to be wet with a solution of fiften per cent. carbolated alcohol, and laid on the abdomen and labia, after having syringed out the vagina with the same.

*Sept. 21st*, 10 A. M.—Found patient still suffering considerably; pulse 140; temperature 102°; respiration labored and short, with intellection impaired, as at first visit; skin hot and dry.

*Sept. 21st.* 5 P. M. — Patient easier, but still suffering much pain, notwithstanding the amount of sedative she had taken; pulse 135; temperature 102°; respiration easier, and with moist rales. Continued same medication, and catheterized patient, obtaining about six ounces of thick, turbid urine. Ordered chloral hydrate, twenty grains, at bed-time, to be given in two doses, and to be followed, in one hour, by morph. sulph., one grain, if quiet was not produced.

*Sept. 22d*, 9 A. M. — Patient slept several hours during the night, and seems much refreshed; pulse, 120, and soft and less throbbing; mind clearer and more hopeful; temperature, $101\frac{1}{2}$°; respiration, easier and free.

Used warm saponaceous enema: procured free movement of the bowels; ordered the following:

℞.—Carbol. acid, ℥ j.
Alcohol, ℥ iv.
Glycerine, ℥ iv.
Aqua, pure, ℥ x.
M. S.—Wash out vagina daily.

Catheter procured good quantity of urine, less turbid, and of less offensive smell than that voided on the day previous.

*Sept. 22d*, 8 P. M. — Found pulse more full, and bowels tympanitic, with much pain, and considerable mental uneasiness. Gave pulv. opii, two grains, and pulv. camph., five grains. Owing to case of labor in the country, did not see my patient again until

*Sept. 23d*, 2 A. M. — although had been sent for several times during the night. Found patient delirious, with pain; pulse, rapid and thready; temperature increased; and respiration hurried and gasping. Was at a loss to know what was the cause of these untoward symptoms.

Ascertained that patient had lifted her babe over herself in bed, and that immediately pain commenced. Examination showed uterus crosswise on the floor of the pelvis; os at sacrum; and body at pubis, pressing against the bladder.

Replaced uterus, and washed out vagina with strong solution of tannin, and gave subcutaneous injection of morph. sulph., one grain; aconitia, one-tenth grain.

*Sept.* 23*d*, 4 P. M.—Patient rational and easier; but not narcotized in the least by the heroic treatment. Has slept considerably during the day; pulse 110, and soft; skin moist; temperature 100°; tongue red, but moist.

*Sept.* 24*th*, 10 A. M.—Patient easier, and fever nearly all gone; pulse 100, and soft; patient sweating profusely. Catheterized patient, obtaining about one pint of high-colored urine. Gave oleaginous enema: produced good stool; gave a powder of one-half grain morph. and three grains quinia, every two hours, alternating with

℞.—Spts. ether. nit., ℥ ij.
Liq. ammon. acet., ℥ j.
Tinct. opii camph., ℥ j.
M. S.—Teaspoonful.

*Sept.* 25*th*.—Patient doing nicely, and pulse coming down: 95, and soft; perspiration free, but not too profuse; temperature normal; with secretions nearly natural.

*Sept.* 26*th*.—Patient able to pass water without catheter, and takes some nourishment with relish.

From this time on, the patient made a slow recovery, on account of the extensive sloughing of the labia and perineum; but by the 15th of October, the patient had so far recovered that I advised an operation for the restoration of the perineum.

The patient objecting to the use of an anæsthetic, and also to counsel, I proceeded, with the help of a good, sensible woman as an assistant, to operate in the following manner:

After having carefully pared the edges of the cicatrized wound, I introduced five silver sutures, bringing them over a piece of elastic catheter, on either side of the wound, drew them well up, and clamped them with lead clamp; and, strange to say, the patient never uttered a word of complaint until the last thing was finished, when the *grit*, which had hitherto sustained her through all, gave way in a copious flood of tears.

Placing the patient on her back, and introducing a glass catheter, with drainage-tube attached, I left patient on a tonic of iron and quinia; and in seven days the wound was healed, so that the catheter was dispensed with altogether; and, on the ninth day from the operation, the sutures were removed, and patient made a very rapid recovery.

I report this case, not for any originality or skill in the surgery of the case, but to show the power of endurance of a frail woman; and, primarily, to urge heroic doses, and prompt, energetic treatment, in cases of peritoneal inflammation.

We, as a profession, are too prone to give minimum doses, and to pander too much to the taste and disposition of patients, even in grave cases.

---

THE difficulty of getting rid of enuresis in young people is sometimes very great. In regard to remedial measures, Dr. Brugelman, led by an article in the *Berlin Klin. Wochenschrift*, resorted to syrup of the iodide of iron, frequently through the day, with every success.

